# Aerobic Physical Exercise as a Neuroprotector Strategy for Ethanol Binge-Drinking Effects in the Hippocampus and Systemic Redox Status in Rats

**DOI:** 10.1155/2019/2415243

**Published:** 2019-07-04

**Authors:** Dinair Pamplona-Santos, Kátia Lamarão-Vieira, Priscila C. Nascimento, Leonardo Oliveira Bittencourt, Márcio G. Corrêa, Savio M. dos Santos, Sabrina C. Cartágenes, Luanna Melo Pereira Fernandes, Marta C. Monteiro, Cristiane S. F. Maia, Rafael Rodrigues Lima

**Affiliations:** ^1^Laboratory of Functional and Structural Biology, Institute of Biological Sciences, Federal University of Pará (UFPA), Belém, PA, Brazil; ^2^Laboratory of In Vitro Tests, Immunology and Microbiology, Institute of Health Sciences, Federal University of Pará (UFPA), Belém, PA, Brazil; ^3^Laboratory Pharmacology of Inflammation and Behavior, Institute of Health Sciences, Federal University of Pará (UFPA), Belém, PA, Brazil

## Abstract

The heavy and episodic EtOH drinking pattern, equivalent to weekend consumption, characterizes the binge-drinking pattern and promotes a misbalance of encephalic metabolic functions, concurring to neurodegeneration and cerebral dysfunction. And for being a legal drug, it has global public health and social relevance. In this way, we aimed to investigate the effects of physical training, in a treadmill, on the deleterious effects of EtOH on hippocampal functions, related to memory and learning. For this, we used 40 Wistar rats, divided into four groups: Control group, Trained group (trained animals with doses of distilled water), EtOH group (nontrained animals with doses of 3 g/kg/day of EtOH, 20% *w*/*v*), and Trained+EtOH group (trained animals exposed to EtOH). The physical exercise was performed by running on a treadmill for 5 days a week for 4 weeks, and all doses of EtOH were administered through intragastric gavage in four repeated cycles of EtOH in binge. After the experimental period, the animals were submitted to the object recognition task and Morris water maze test, and after being euthanized, the blood and hippocampus were collected for Trolox Equivalent Antioxidant Capacity (TEAC), Reduced Glutathione Content (GSH), and Nitrite and Lipid Peroxidation (LPO) level measurements. Our results showed that EtOH caused marked oxidative stress and mnemonic damage, and the physical exercise promoted neuroprotective effects, among them, the modulation of oxidative biochemistry in plasma (by restoring GSH levels) and in the hippocampus (by reducing LPO levels and increasing antioxidant parameters) and cognitive function improvement. Therefore, physical exercise can be an important prophylactic and therapeutic tool in order to ameliorate and even prevent the deleterious effects of EtOH on cognitive functions.

## 1. Introduction

Ethanol (EtOH) is a psychotropic drug that generates behavioral changes and may lead to addiction, i.e., dependency. It is a licit substance, with easy access and even encouraged by society, but excessive consumption is associated with psychosocial and medical disorders, being considered as a serious public health issue, both in terms of morbidity and mortality [1].

Furthermore, EtOH has been associated with short- and long-term neuropsychological effects, and the increased prevalence of the binge-drinking pattern during adolescence, when the brain is still in development and maturation, constitutes an important global health problem since it predisposes individuals to dependence and comorbidities [2–4]. This is quite evident in the hippocampus, where the consumption of EtOH in the intermittent model promotes the reduction of neurogenesis, hippocampal volume, synaptic communication, and neurotrophins associated with neuroplasticity as a brain-derived neurotrophic factor (BDNF) [2, 5–8], which is therefore strongly associated with cognitive impairments.

Considering this problem, several strong strategies for neuroprotection have been studied in experimental animal models and humans. Physical exercise seems to be associated with reduction of neuroinflammation [9–11], improvement of cognitive functions [12–14], increase in BDNF levels [15–17], hippocampal neurogenesis modulation [10, 13, 16], cerebral oxidative stress modulation [18, 19] and induction of several positive morphological changes [10, 15, 17]. However, the beneficial effects of the association between physical exercise and EtOH consumption are not completely understood, still requiring elucidation of the main mechanism by which physical training may help alcoholic drinkers, especially over cognitive functions associated with hippocampal formation.

In this perspective, the objective of this study was to investigate the effects of aerobic physical exercise of moderate intensity on the possible neuroprotection and/or minimization of the alcoholic intoxication damage in the hippocampus of rats.

## 2. Materials and Methods

### 2.1. Ethical Statement and Experimental Group Formation

This research was submitted to the Ethics Committee on Experimental Animal Research (CEPAE) from the Federal University of Pará (UFPA) and authorized under the protocol CEPAE–UFPA 227-14l, following all NIH guidelines for the use and care of experimental animals [20]. Forty male Wistar rats (*Rattus norvegicus*), weighing between 60 and 80 g and 30 days old, were provided from the UFPA animal house and placed in a collective with 4 animals each. During the experimental period, the animals were housed in a climate-controlled (25°C) room with a dark-light cycle of 12 h, respectively (lights on at 7 a.m.), and water and food *ad libitum*.

**Figure 1:**
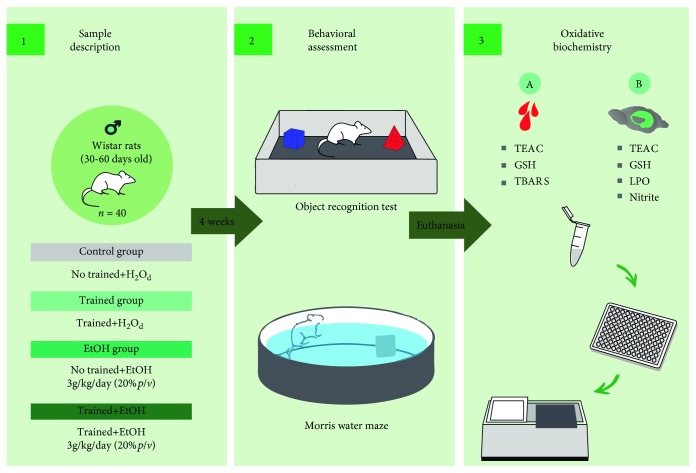
Sample description and experimental design. (1) Sample characteristics and the division of the experimental groups of the treadmill physical training and ethanol (EtOH) or distilled water (H_2_O_d_) administration; (2) after four weeks (28 days), accomplishment of the behavioral assays: object recognition test and Morris water maze; (3A) after euthanasia, blood plasma collection for oxidative balance analyses through Trolox Equivalent Antioxidant Capacity (TEAC), Reduced Glutathione [14], and Thiobarbituric Acid Reactive Substances (TBARS); (3B) also, hippocampus collection for oxidative balance analyses through TEAC, GSH, Lipid Peroxidation (LPO), and nitrite levels (Nitrite).

The experimental animals were divided into four groups: Group 1, composed of sedentary animals treated only with distilled water by intragastric gavage (Control group); Group 2, trained animals treated only with distilled water by gavage (Trained group); Group 3, sedentary animals treated with EtOH by intragastric gavage (EtOH group); and Group 4, trained animals treated with EtOH by intragastric gavage (Trained+EtOH group).  Figure 1 summarizes all methodological steps of this study.

### 2.2. Physical Training Protocol

The physical training protocol was performed in a treadmill (Insight, Brazil) adapted for rodents, during four consecutive weeks for 30 minutes each training session [21–23]. The running sessions were executed between 8 a.m. and 12 a.m. in a motorized treadmill adapted for rodents, measuring a width of 10 cm and a length of 50 cm and with bays separated by acrylic walls.

### 2.3. Protocol of EtOH Exposure

**Figure 2:**
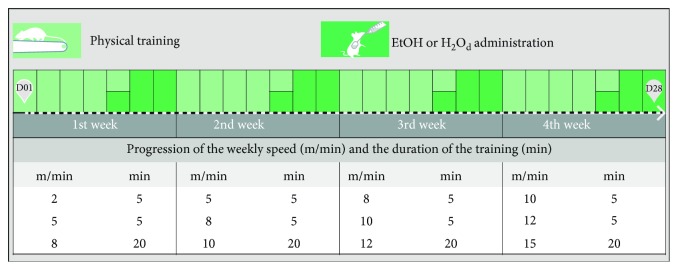
Experimental schedule of the treadmill physical training protocol and ethanol (EtOH) or distilled water (H_2_O_d_) administration by intragastric gavage since day 1 (D01) until day 28 (D28). The physical training protocol was adapted from Arida et al. [21].

Through intragastric gavage, we administered distilled water or ethanolic solution, at a dose of 3 g/kg (20% *w*/*v*), being weekly adjusted after weighting the animals [24, 25]. At the fifth day of training in each week, during three consecutive days in the week, we performed the water or EtOH administration only after the last training session of the day. In this way, the animals went through twelve EtOH exposures throughout the experimental period.  Figure 2 summarizes the training and EtOH exposure protocols.

### 2.4. Behavioral Assessment

The tests were performed 24 hours after the last administration of EtOH or distilled water, and 10 animals per group were randomly selected and conducted to the assay room, where the sound and illumination were controlled in order to avoid any stressful environment.

#### 2.4.1. Object Recognition Test

This task investigates the emotionality and memory capacity of the animals. The apparatus for this assay consists of a square wooden arena (100 × 100 × 30 cm), with a recording camera on the roof, in which the videos recorded are further analyzed by ANY-maze software (Stoelting Co., UK). The task consists of four phases: habituation (30 minutes in the arena), training and two test phases, in which two objects are placed in extreme corners of the arena. In the training phase, the animals are presented to two objects that they will become familiar with for 3 minutes, while in the test phase, one of the objects is replaced by a different one from that which was already familiar to the animals. In this way, the investigation time spent by the animals on each object in the training phase (T1) was recorded, as well as the time spent exploring the newest object in the test phase (T2) by the camera mentioned before. The exploration of an object was defined as the head of the animal facing the object at a distance equal to or less than 4 cm [26]. The analyses were performed considering the total exploration time spent on the two objects in the training phase, and the recognition index was defined by the difference in the time of exploration between the new object and the familiar object divided by the total time spent exploring between the same objects in the test phases: (T2 − T1)/(T2 + T1).

#### 2.4.2. Morris Water Maze Test

The spatial memory was verified by the Morris water maze [27]. The apparatus consists of a circular water tank (diameter of 150 cm), with an acrylic platform underwater and a recording camera positioned on the roof. The tank was dived into four quadrants (Q1-Q4) by imaginary lines, and it was filled with water ±25°C up to 45 cm and colored with a blue nontoxic and water-soluble dye to turn it dark (to contrast with the animal color in the recordings), and on Q4, we positioned the acrylic platform with a diameter of 10 cm and a height of 43 cm. It used a version of reference spatial memory, in which the experimental protocol consists of four training sessions and two test sessions, as previously described by Prediger [28]. The first test session (short-term memory) was executed 1 hour after the training, and 24 hours after, the test to evaluate long-term memory was proceeded with the same methodology [28, 29].

### 2.5. Blood and Hippocampal Oxidative Biochemistry Analyses

Ten animals were randomly selected and used for evaluation of the oxidative biochemistry state in the blood and hippocampus. The animals were deeply anesthetized through intraperitoneal injection of ketamine hydrochloride (90 mg/kg) and xylazine hydrochloride (10 mg/kg), and then, the blood collection was executed by intracardiac puncture in tubes containing EDTA. The blood samples were centrifuged for 10 minutes at 1400 rpm, and the plasma was collected and stored at -80°C. The hippocampi were collected after total loss of retinal and paw reflexes by craniotomy and brain dissection. The hippocampi were washed in PBS and immediately frozen in liquid nitrogen and stored at -80°C until further analyses. For biochemical analyses, firstly, the hippocampus samples were thawed, suspended in Tris Buffer Solution (HCl 20 mM, pH 7.4) at 4°C, and homogenized by ultrasonic degradation, and after, the homogenate was centrifuged at 3000 rpm for 10 minutes (at 4°C), in which the supernatant was collected for the analyses described below.

#### 2.5.1. Trolox Equivalent Antioxidant Capacity (TEAC)

This method was described by Rufino et al. [30] and consists of [31] the 2,2-azino-bis(3-ethylbenzothiazoline)-6-sulfonic acid (ABTS; 7 mM) incubation with potassium persulfate (2.45 mM) at room temperature for 16 hours to produce the radical ABTS^+^. The work solution was prepared from the ABTS^+^ radical in PBS (pH 7.2) until absorbance of 0.7 ± 0.02 at 734 nm. Subsequently, an aliquot of 35 *μ*L from the samples or trolox standard was added to 2970 *μ*L of ABTS solution, and the absorbance was read after 5 minutes. The absorbances were read in triplicate and we established a standard curve in order to calculate the proportional TEAC [32]. The results were expressed as percentage of control.

#### 2.5.2. Glutathione Peroxidase (GPx) and Glutathione Reductase (GR) Assay

The assay of GPx was based on the method described by Flohe and Gunzler [33]. One unity of enzyme is defined as the quantity of enzyme that catalyzes the oxidation of 1 *μ*mol of NADPH per minute. The enzymatic activity was determined using the extinction coefficient of 6.2 M^−1^ cm^−1^. The blanks were made in the absence of enzymatic extract and in the absence of GSSG. The GR activity was executed following the oxidation of 0.1-0.25 mM of NADPH by 1-5 mM GSSG in 1 mL of potassium phosphate buffer (50 mM, pH 7.2), with 0.5 mM of EDTA, containing 50 *μ*L of protein extract [34]. The oxidation of NADPH was monitored at 340 nm. We also used the methodology described by Smith et al. [35] that uses 5,5′-dithiobis(2-nitrobenzoic acid) (DTNB; 0.47 mmol). The results were expressed as a percentage of control.

#### 2.5.3. Lipid Peroxidation (LPO) Determination by Thiobarbituric Acid Reactive Substances

This procedure is a method that evaluates LPO and acts as an indicator of oxidative stress. It is based on the reaction of MDA and other substances with thiobarbituric acid (TBA), performed according to the proposed method in da Silveira et al. [36]. In each assay tube, 10 nM of TBA (Sigma-Aldrich) and 0.5 mL of sample were added. After, the tubes were heated at 94°C for 60 minutes to form the complex MDA-TBA, which is dyed pink. After this procedure, the samples were refrigerated in tap water and the butyl alcohol was added to the samples in order to obtain maximum extraction of MDA in the organic phase. Finally, the tubes were centrifuged, and the supernatant was collected and read at 535 nm. The results were expressed as a percentage of control.

#### 2.5.4. Estimation of Nitrite Level Assay

For nitrite level estimation, we used Griess' protocol [37] that consists of centrifuging the samples at 21000g during 20 minutes at 4°C and using the supernatant to proceed the assay. The samples were incubated at room temperature during 20 minutes with 100 *μ*L of Griess reagent (0.1% naphthyl-ethylenediamine and 1% sulfonamide in 5% phosphoric acid—1 : 1). The absorbances were read at 550 nm by a spectrometer, and we established a standard curve by the absorbance of known concentrations of nitrite. The results were plotted and expressed as a percentage of control.

#### 2.5.5. Statistical Analyses

After data collection, the distribution was tested by the Shapiro-Wilk method for verification of normality. Statistical comparisons between groups were performed using one-way ANOVA and Tukey post hoc test, except for the weight curve that was evaluated with two-way ANOVA followed the Tukey post hoc test. The *p* values < 0.05 were considered statistically significant. The GraphPad Prism 7.0 (San Diego, CA, USA) software was used to perform statistical analyses.

## 3. Results

### 3.1. The Repeated Cycles of EtOH in a Binge-Like Pattern and Treadmill Physical Exercise Did Not Affect the Animals' Weight Gain

**Figure 3:**
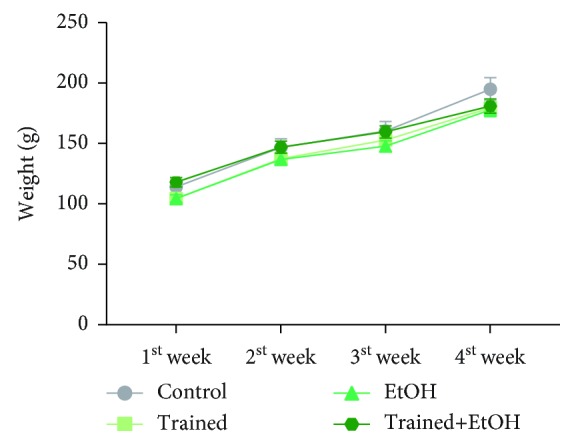
Effects of four cycles of treadmill physical training and exposure to binge-like ethanol, for 28 days, on body weight gain (g) of Wistar rats. Results are expressed as mean ± standard error of the mean. Two-way ANOVA and Tukey's post hoc test, *p* > 0.05.

Repeated cycles of physical training on the treadmill and EtOH binge drinking for four weeks did not interfere with the animals' weight (*p* = 0.937). At the end of the experiments, the animals did not show mean body weight difference (Control group: 194.9 ± 9.5; Trained group: 179.5 ± 4.6; EtOH group: 177.86 ± 5.59; Trained+EtOH group: 180.93 ± 5.85) as observed in Figure 3.

### 3.2. The Aerobic Physical Exercise Modulated the Oxidative Biochemistry of Rats' Plasma by Reestablishing Glutathione Levels after 4 Weeks of EtOH Exposure in a Binge-Like Manner

**Figure 4:**
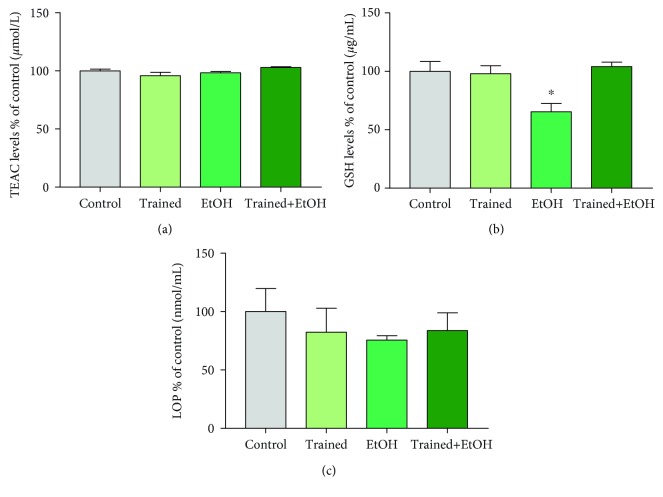
Effects of four cycles of treadmill physical exercise and exposure to binge-like ethanol, for 28 days, on oxidative balance in the blood plasma of Wistar rats. (a) TEAC levels, (b) GSH levels, and (c) Lipid Peroxidation (LPO). Results are expressed as mean ± standard error of the mean of control percentage. One-way ANOVA and Tukey's post hoc test, *p* < 0.05. ^∗^Statistical difference in relation to the other groups.

After repeated cycles of physical exercise on the treadmill and EtOH binge-like exposure for four weeks, EtOH did not induce changes in TEAC levels (EtOH group: 93.35 ± 1.05%; Trained+ EtOH group: 102.9 ± 0.65), when compared to plasma from control and trained-only animals (Control group: 100 ± 1.57%; Trained group: 95.77 ± 3.01%; *p* = 0.076; Figure 4(a)).

However, we observed a significant decrease in GSH plasma levels in rats exposed to EtOH (EtOH group: 65.37 ± 7.13%) compared to the other groups (Control group: 100 ± 8.38%; Trained group: 97.93 ± 6.85%; Trained+EtOH group: 104 ± 3.87%; *p* = 0.005; Figure 4(b)), emphasizing that physical exercise avoided the changes induced by EtOH. No significant difference was observed in TBARS levels among the experimental groups (Control group: 100 ± 19.86%; Trained group: 82.26 ± 20.7%; EtOH group: 75.55 ± 3.77%; Trained+EtOH group: 83.74 ± 15.16%; *p* = 0.965; Figure 4(c)).

### 3.3. The Aerobic Physical Training Modulated the Oxidative Biochemistry Balance in the Hippocampus of Rats Exposed to Four Cycles of Binge Drinking

**Figure 5:**
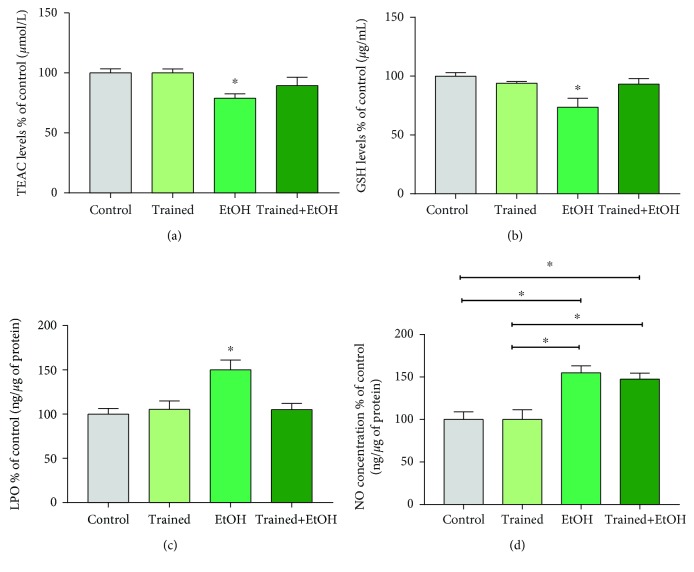
Effects of four cycles of the treadmill physical exercise and exposure to binge-like ethanol, for 28 days, on oxidative balance in the hippocampus of Wistar rats. (a) TEAC levels, (b) GSH levels, (c) percentages of Lipid Peroxidation (LPO) in relation to the control group, and (d) percentages of nitrite per milligram of protein in relation to the control group. Results are expressed as mean ± standard error of the mean. One-way ANOVA and Tukey's post hoc test, *p* < 0.05. ^∗^Statistical difference in relation to the other groups or between the groups indicated.

As observed in Figure 5, the exposure to EtOH in a binge-like pattern also misbalanced the oxidative biochemistry in the hippocampus of rats. The EtOH reduced TEAC levels (EtOH group: 78.88 ± 3.67%; Figure 5(a)) in comparison to the control group (100 ± 3.41%; *p* = 0.016). We observed that the physical exercise could avoid this misbalance provoked by EtOH (Trained+EtOH group: 89.38 ± 7.09%; *p* = 0.385; Figure 5(a)).

Exposure to EtOH also modified oxidative parameters related to GSH levels (EtOH group: 73.58 ± 7.54%) when compared to the control group (100 ± 2.87%; *p* = 0.009) that was not observed in the trained animals (Trained group: 93.95 ± 1.44%; Trained+EtOH group: 93.24 ± 4.7%; *p* > 0.05; Figure 5(b)).

Furthermore, an increase of LPO was observed in the hippocampus of animals exposed to EtOH (EtOH group: 150 ± 11.09%) in comparison to the other group, highlighting the reestablishment of the LPO levels to normal levels due to physical exercise (Control group:100 ± 6.33%; Trained group:105.4 ± 9.44%; Trained+EtOH group:105 ± 7.03%; *p* = 0.003; Figure 5(c)).

Besides that, the EtOH group presented higher nitrite concentrations (EtOH group: 155 ± 8.24%) in comparison to the other groups without EtOH exposure (Control group: 100 ± 9.04%; Trained group: 100 ± 11.55%; *p* < 0.05). However, there was no statistical difference in comparison to the Trained+EtOH group (147.5 ± 7.08%; *p* = 0.958; Figure 5(d)).

### 3.4. Physical Exercise Minimized Memory Deficits of Rats Exposed to Repeated Cycles of EtOH in a Binge-Like Pattern

**Figure 6:**
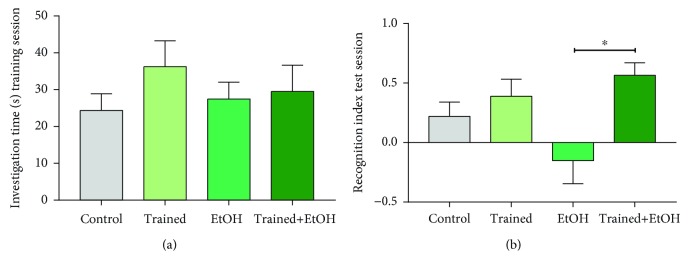
Effects of four cycles of the treadmill physical exercise and exposure to binge-like ethanol, for 28 days, on working memory of Wistar rats. Results are expressed as mean ± standard error of the mean of (a) investigation time (s) in the training session and (b) recognition index in the test session. One-way ANOVA and Tukey's post hoc test, *p* < 0.05. ^∗^Statistical difference in relation to the other groups or between the groups indicated.

Repeated cycles of EtOH binge-like consumption for four weeks induced injury to working memory, long-term spatial memory, and learning ability in rats, as observed in the object recognition and MWM tests. In the object recognition test, trained animals that were exposed to EtOH showed better recognition index (Trained+EtOH group: 0.56 ± 0.1) when compared to those which were only exposed to EtOH (EtOH group: −0.15 ± 0.19; *p* = 0.005; Figure 6), revealing the benefits of physical exercise on short-term memory of animals exposed to EtOH.

**Figure 7:**
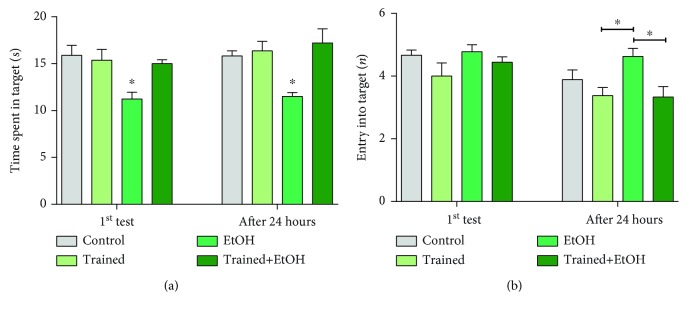
Effects of four cycles of the treadmill physical exercise and exposure to binge-like ethanol, (EtOH) for 28 days, on spatial long-term memory and learning capacity of Wistar rats. Results are expressed as mean ± standard error of the mean of (a) time spent in the target (s) and (b) number of entries into the target. Both measurements were performed in the first test and the test after twenty-four hours. One-way ANOVA and Tukey's post hoc test, *p* < 0.05. ^∗^Statistical difference in relation to the other groups or between the groups indicated.

When learning and memory were assessed by the Morris water maze (Figure 7), our data showed that the physical exercise avoided the deleterious effects of EtOH. This fact was observed in the first test (Control group: 15.89 ± 1.06; Trained group: 15.38 ± 1.14; EtOH group: 11.22 ± 0.74; Trained+EtOH group: 15 ± 0.42; *p* = 0.002) and on the time spent in the target quadrant during the test (Control group: 15.83 ± 0.54, Trained group: 16.38 ± 1.01, Trained group: 11.5 ± 0.42, Trained+EtOH group: 17.2 ± 1.53, *p* = 0.002) (Figure 7(a)). Regarding the number of entries in the target quadrant (Figure 7(b)), there was no difference in the first test among the groups (Control group: 4.66 ± 0.16; Trained group: 4 ± 0.42; EtOH group: 4.77 ± 0.22; Trained+EtOH: 4.44 ± 0.17; *p* = 0.182), while in the second test, the EtOH group showed difference in comparison to the Trained and Trained+EtOH groups (Trained group: 3.37 ± 0.26; EtOH group: 4.6 ± 0.26; Trained+EtOH group: 3.33 ± 0.33; *p* < 0.05), but not in comparison to the control group (Control group: 3.88 ± 0.3; *p* = 0.319).

## 4. Discussion

Considering the health impacts that EtOH consumption may cause and the constant need to investigate new therapeutic tools that ameliorate its damage, this work brings important data about how physical exercise positively affects cognitive functions that are deeply affected by EtOH consumption, even in a binge-drinking pattern. This study revealed that physical exercise is associated with GSH level restoration in the blood of rats exposed to EtOH. Moreover, this nonpharmacological therapeutic tool is also associated with TEAC and GSH level restoration in the hippocampus of rats exposed to EtOH and also the reduction of LPO levels into the basal state, comparable to nonexposed animals. And following this perspective of EtOH binge-drinking pattern impacts, we are showing that physical exercise substantially improves cognitive hippocampal functions, such as memory and learning.

Binge drinking or episodic heavy drinking is the practice of consuming large amounts of alcohol in a single session and causing a blood alcohol concentration (BAC) equal to or greater than 0.8 g/L. This dosage is equivalent to five or more doses for men, or four or more doses for women in a two-hour period [38]. The model of alcoholic intoxication in the binge-drinking pattern used in this work reproduces the pattern of encephalic oxidative damage seen in humans who drink in binge [39–42]. However, the degree of severity of brain damage may depend on some variables, such as exposure time, sex, and age.

The exposure to EtOH is associated with several neurological disorders, which may include prenatal exposure, featuring fetal alcohol syndrome, and effects in postnatal individuals, like poor motor performance and cognitive decline [2, 39, 43, 44]. This last one is commonly associated with visuospatial capacities, executive functions, and episodic memory [45, 46]. Following this cognitive component, the hippocampus plays a pivotal function in this process, since, anatomically, it is related to other structures that are deeply associated with memory processes [47]. Also, the hippocampus is an integrator center of projections from the perirhinal, parahippocampal, and entorhinal cortices [48], which are included in communication pathways for spatial memory (part of declarative memories) [49–51]. And although the literature presents controversial findings of the hippocampal role in the object recognition task, some studies have pointed that this organ is mainly associated with visual memory and affects the recognition memory; therefore, it also reinforces the hippocampus as an associative structure [52–58] that is required for an efficient cognitive process. In this way, for our behavioral assessment, we elected both these paradigms to investigate hippocampal failure after EtOH exposure: spatial memory and recognition memory.

The object recognition test is based on the presentation of the rodents to familiar and new objects. Instinctively, they spend most of their time exploring the new object. This preference is used as an indication of memory in relation to the familiar object [59]. Our results in this test were markedly significant, since rats exposed to EtOH had marked memory deficits compared to controls and trained exposures. The Morris water maze test, especially indicated for measuring spatial learning and hippocampal-dependent mnemonic processes [27–29, 60], demonstrated that the rats exposed to EtOH had long-term spatial memory deficit.

This study showed that alcoholic intoxication in a binge pattern for three consecutive days per week (during four weeks), generated oxidative stress in the hippocampus but not in the blood. Hippocampal stress, on the other hand, promoted short-term and long-term memory impairment. In addition, for the first time, it has been shown that forced physical exercise, on a treadmill, concomitantly with EtOH intoxication, minimized the deleterious oxidative and behavioral effects generated by exposure to EtOH in young adult rats.

Several studies have highlighted the strong association between oxidative stress and cognitive decline, specially evaluating anti- and prooxidative agents in the blood of patients with compromised cognitive functions [61–64]. And interestingly, those studies have pointed that reduced levels of glutathione peroxidase or glutathione in the blood may be a strong biomarker for cognitive decline [61, 63, 64]. In this perspective, we hypothesize that the reduction of GSH levels found in the blood of rats exposed to EtOH may exert a strong association with the hippocampal deficit found in this study. Despite repeated cycles of intoxication in binge, the serum concentration of TBARS was not significant among the groups, discarding the possibility of physical exercise playing a harmful role in the oxidative dynamics. Still, in a recent work [40], the authors observed that adolescent female Wistar rats, treated with EtOH in a binge pattern, presented cumulative effects on oxidative damage in the motor cortex and liver, which were also not detectable peripherally; it that seems that gender and age variables did not interfere in this scenario.

We did not notice significant changes in the body weight of our rats among the groups and throughout the experiment. Previous work reported that EtOH induced a decrease in body weight. This decrease seems to be dose-dependent, and the higher the EtOH dose, the lower the weight of rats [39, 65]. This decrease was significant from the dose of 1.2 g of EtOH per kilogram of body weight over four weeks [65]. Our sample's weight remained stable for the same exposure period at the dose of 0.8 g/kg. Physical exercise also did not interfere with weight loss or gain in our experimental design.

When in the blood, EtOH molecules are transported quickly to all tissues that have cells with high concentrations of water like the brain, liver, heart, and kidneys [66]. The vulnerability of the CNS to the effects of EtOH and exposure during brain development may cause irreversible abnormalities of brain structures and functions [3, 4]. The evaluation of LPO in the CNS system has a relevant importance considering that the brain is particularly vulnerable to free radicals, due to the high oxygen consumption and high content in easily oxidizable substrates, in contrast to the low activity of antioxidant enzymes.

The CNS has a high cellular metabolism, which demands high consumption of oxygen and naturally results in ROS production, which requires an efficient antioxidant system acting [67]. EtOH consumption promotes oxidative imbalance causing LPO, DNA, and protein oxidation, which is enough to promote mitochondrial dysfunction and subsequent neuronal damage. On the other hand, ROS is associated with neuroinflammation that also contributes to the alteration of organelles; however, detoxification, repair, and adaptation are expected responses during an inflammatory state, which, in a deficit situation, may drive to oxidation of the cellular components mentioned above [67, 68], which may increase cell death resulting in cognitive impairments such as memory and learning [69] also observed in this study.

Therefore, the toxicity of EtOH during its metabolism leads to a marked oxidative stress, including in the CNS, due to a reduction of antioxidant enzymes and marked production of oxidizing molecules, such as H_2_O_2_, hydroxyl radicals (OH^·^), and singlet oxygen during the process to the mitochondrial transport chain phosphorylation [70, 71]. Recently, our group showed that EtOH exposure is able to promote oxidative changes and functional changes in the cerebellum of rats, which can be minimized by physical exercise due to the neuroprotection and decrease in total antioxidant capacity (TEAC) and an increase in levels of Lipid Peroxidation (MDA) in the cerebellum [23]. In addition, we also showed that EtOH exposure led to oxidative stress with increased MDA levels, possibly reflecting on the CNS, which led to a profile of psychiatric disorders or cognitive impairment and impact motor performance [2, 71]. In this regard, we also showed that this EtOH consumption induces marked oxidative stress in the liver and motor cortex leading to changes in motor function; however, it did not alter the levels of prooxidant and antioxidant factors in the blood similar to the results demonstrated in this study [72]. In this study, our experimental data showed that MDA levels were not altered in the periphery, indicating that in clinical practice, MDA levels in the blood could not be used as peripheral markers of ethanol toxicity. These findings may be explained by the fact that reduced antioxidant defenses against oxidative damage may particularly affect the organs most susceptible to this type of damage, such as the brain. Brain cells are more vulnerable to oxidative damage due to reduced levels of antioxidant enzymes and a high level of oxidative metabolism in this tissue [73]. Thus, in this tissue, ROS can lead to oxidative damage in cellular components, impairing cellular energy and signaling pathways (redox signaling) that can cause acute and chronic CNS dysfunctions [74].

In contrast to EtOH, the beneficial effects of exercise on brain functions are well documented, including by our group, who recently showed that moderate-intensity aerobic exercise improved antioxidant levels and reduced oxidative stress in regions associated with motor functions, as the motor cortex, cerebellum, and striatum [23, 69]. In addition, physical exercise may modulate mitochondrial functions, elevating autophagy and expression of neurotrophic factors such as BDNF (brain-derived neurotrophic factor), FGF (fibroblast growth factor), and VEGF (vascular endothelial growth factor) by the development and maintenance of the nervous system [15, 75–78]. Thus, increased levels of BDNF and other transcription factors, such as the transcription factor cAMP response element-binding protein (CREB), can help to sustain the structural and functional integrity of the hippocampus and neurogenesis and synaptic processes such as long-term potentiation (LTP) [75].

Accordingly, in damage caused by EtOH in the CNS, several studies showed that exercise can increase the availability of neurotrophins, leading to a sufficient supply of trophic molecules, so that both mature cells and those newly generated in the brain become more resistant to the damage caused by alcohol [15, 76–78]. In addition, physical exercise alters levels of neurotransmitters in the hippocampus, such as acetylcholine by stimulating cholinergic fibers that lead to increased vasodilation, and thus elevate blood flow in the hippocampus during exercise [79]. Thus, these effects caused by physical exercise have an essential role in the performance of hippocampus-dependent learning and memory tasks and stimulate the process of redox regulation, thus controlling the oxidative stress caused by alcohol [75]. These findings may explain our data that show the beneficial effects of exercise on behavioral impairments and changes in redox status induced by EtOH, such as increased LPO and reduced levels of GSH and TEAC in the hippocampus. Thus, the results suggest that EtOH induces oxidative stress that exerts pathological effects on the hippocampus and on cognitive functions, and this process can be regulated and thus controlled by physical exercise.

In this regard, the protocol of physical exercise used [21] was absolutely innocuous in relation to oxidative dynamics, since our biochemical analyses in the plasma show that the levels of the marker of LPO were not significant when we compared the trained group with the control one and the decrease of the levels of GSH was seen only in the untrained intoxicated group. In recent work [80], the authors also used a forced exercise protocol on a treadmill and showed that exercise increased the level of cortisone and correlated with increased ischemic brain damage, although they did not evaluate oxidative damage in the blood tissue. This effect, unlike ours, can be explained by the difference between the exercise protocols. Daily, we kept the animals for at least 10 minutes at lower speeds and the remaining 20 minutes at the highest velocity of the weekly program (see Figure 1). Treadmill exercise protocols that standardize a gradual increase in velocity allow the maintenance of the balance between pro- and antioxidants as confirmed by our results.

We must consider the paradox in relation to the effects of physical exercise practice, as it causes an increase in oxygen consumption and, consequently, the generation of reactive oxygen species (ROS). However, the increased synthesis of antioxidant enzymes that are also induced by physical exercise constitutes a metabolic adaptation capable of protecting cells and tissues from the oxidative stress imposed by physical exercise itself [14, 81], as previously mentioned. The elevation of LPO in the hippocampus of rats exposed to EtOH and the elevation of TEAC in Trained+EtOH rats translate this metabolic adaptation and allow us to suggest that the cognitive improvement we observed was due to this oxidative biochemistry status reestablishment.

In this way, we strongly believe that one of the mechanisms by which EtOH exerts its deleterious effects on CNS, especially on cognitive functions, is by oxidative stress. But more interestingly is that the moderate and gradual intensity of physical exercise restores the normal rates of antioxidant and prooxidant agents in the blood and/or in the hippocampus.

## 5. Conclusions

We demonstrated that physical exercise is a strong nonpharmacological therapeutic tool for the prevention of cognitive dysfunction caused by EtOH exposure in binge-drinking pattern consumption. The physical exercise exerts a role in reestablishing the redox status by elevating GSH levels in the blood and hippocampus of rats exposed to EtOH; besides, it increases TEAC levels and reduces LPO levels, both in the hippocampus, which is associated with the improvement of cognitive functions of the exposed animals. One of the main contributions of the present study was to show, by a preclinical model, that the damage promoted in the hippocampus of rats due to excessive EtOH consumption can be minimized by moderate and gradual intensity of aerobic physical exercise. In this way, by unraveling the protective mechanisms, the identification of important therapeutic targets become the main strategy for neuroprotection with nonpharmacological alternatives, such as physical exercise.

## Data Availability

The data used to support the findings of this study are available from the corresponding author upon request.
